# Clinical and Genetic Aspects of Alopecia Areata: A Cutting Edge Review

**DOI:** 10.3390/genes14071362

**Published:** 2023-06-28

**Authors:** Chih-Yi Ho, Chiu-Yen Wu, Jeff Yi-Fu Chen, Ching-Ying Wu

**Affiliations:** 1 Department of Dermatology, College of Medicine and Post Baccalaureat Medicine, Kaohsiung Municipal Ta-Tung Hospital, Kaohsiung Medical University Hospital, Kaohsiung Medical University, Kaohsiung 801, Taiwan; 2 Department of Cosmetic Science, Chang Gung University of Science and Technology, Taoyuan 333, Taiwan; 3 Department of Biotechnology, Kaohsiung Medical University, Kaohsiung 807, Taiwan

**Keywords:** molecular genetics, alopecia areata, alopecia totalis, alopecia universalis, genome-wide association study (GWAS), DNA genotyping, JAK-STAT signaling pathway, human leukocyte antigen (HLA), single nucleotide polymorphism (SNP)

## Abstract

Alopecia areata (AA) is a chronic, non-scarring, immune-mediated skin disease that affects approximately 0.5–2% of the global population. The etiology of AA is complex and involves genetic and environmental factors, with significant advancements in genetic research occurring in recent years. In addition to well-known genes such as *PTPN22*, *CTLA4*, and *IL2*, which have been widely supported as being associated with AA, an increasing number of specific gene-related loci have been discovered through advances in genetic research. For instance, gene analysis of microRNAs can reveal the critical role of miRNAs in regulating gene expression, aiding in the understanding of cellular and organismal functional regulatory mechanisms. Furthermore, numerous studies have confirmed the existence of correlations between AA and other immune-related diseases. Examples include hyperthyroidism and rheumatoid arthritis. By understanding the interrelationships between AA and other immune diseases, we can further comprehend potential shared genetic foundations or pathogenic mechanisms among different diseases. Genetic research plays a crucial role in unraveling the pathogenesis of AA, as the identification of genetic variations associated with AA can assist in formulating more effective and targeted treatment strategies.

## 1. Introduction

Alopecia areata (AA) is an autoimmune disease that leads to hair loss on the scalp and other hairy parts of the body as a result of hair follicles being attacked by the immune system. This disease typically presents as circular or patchy bald spots without scarring. In some cases, it can also affect the nails, making them brittle or pitted [1]. AA is relatively common and can affect people of any age, with a slightly higher prevalence in adult females [2]. The exact cause of AA is unknown, but it is believed to involve genetics, environmental factors, and immune system dysfunction. AA could have a significant negative impact on patients’ quality of life, affecting their psychological health, social life, and overall satisfaction. Patients may experience anxiety, depression, low self-esteem, and social difficulties, all of which can damage their quality of life [3]. In addition, due to the lack of hair protection, the affected areas are more vulnerable to exposure to ultraviolet radiation, leading to sunburn or sunspots on the scalp. Overall, AA can have negative impacts on the appearance, emotions, and social status of the affected individuals, highlighting the importance of early diagnosis and treatment.

Genetic research is crucial for understanding the etiology and treatment of AA. Many studies have shown that AA may result from the abnormal expression and interaction of multiple genes [4,5] which are involved in biological processes such as the immune system and hair follicle growth and development. Thus, genetic testing can help assess patients’ susceptibility and predict and diagnose the disease. For instance, central centrifugal cicatricial alopecia (CCCA) is a prevalent alopecia disorder that predominantly affects women of African and African descent. It has been established that mutations in *PADI3* are implicated in CCCA. If patients exhibit these mutations during genetic testing, they may have an increased predisposition to CCCA or be at risk of developing the condition [6]. Furthermore, AA-based genetic research can facilitate the development of new treatments. Indeed, recent breakthroughs in genetic research have led to the application of JAK inhibitors in the treatment of AA. This review article conducted a literature search on PubMed utilizing key terms including alopecia areata, alopecia totalis, alopecia universalis, genetic, and GWAS, among others and provides an overview of the epidemiology and pathology of and treatment options for AA, as well as a comprehensive review of the genetic research conducted in the field.

## 2. Epidemiology

Currently, the global prevalence of AA is approximately 0.1%, with a lifetime incidence of around 2% [2,7]. Most literature suggests that there is no significant difference in incidence rates between males and females. Some studies have suggested a higher incidence in females, but this may be due to greater awareness and attention to hair loss and subsequent treatment in females, while the incidence also varies across different countries [8]. AA can occur at any age, but the most common age range is between 30 and 40 years old, with some differences in diagnosis age between males and females. Males are more likely to be diagnosed in childhood, while females are more likely to present during puberty [9]. Recent research on AA in children has indicated that it is the third most common skin disease manifestation in children. Between 2010 and 2017, over 140,000 children in the United States were diagnosed with AA, with a prevalence rate of approximately 0.23% and the highest incidence in the 11–12-year age group. In addition, it was found that the number of new cases of AA in children is increasing each year [10]. Here we summarize the incidence and prevalence rates of AA in different countries in Table 1, the prevalence rates by age in Table 2, and the age of diagnosis/onset in Table 3.

## 3. Etiology and Pathogenesis

AA is a complex autoimmune disease with multiple etiological factors. The disease is associated with genetic, environmental, infectious, and immune system abnormalities [5]. Immune system dysfunction is one of the main causes of AA pathogenesis, with abnormal T and B lymphocytes in AA patients secreting cytokines and autoantibodies that attack hair follicle cells, leading to hair loss. These immune cells release cytokines, such as interferon-γ and tumor necrosis factor, when they enter the hair follicle and then induce apoptosis of hair follicle cells [5]. Furthermore, AA is an autoimmune disorder characterized by immune-mediated hair loss, and the currently acknowledged pathogenesis primarily revolves around the disruption of immune privilege. Immune privilege refers to the phenomenon where specific tissues or regions exert inhibitory effects on the immune system to protect themselves from immune attacks. This privileged state is observed in certain tissues or organs, such as the eyes, testes, and placenta. Mechanisms underlying immune privilege include reduced antigen presentation, immune cell death, or the release of immunosuppressive molecules. Disruption of immune privilege can result in immune system attacks on these tissues, leading to diseases or autoimmune reactions [29,30]. Under normal circumstances, hair follicles are recognized as immune-privileged sites by the body’s immune system, remaining unaffected. However, in individuals with AA, the immune system erroneously perceives the hair follicles as exogenous entities, instigating an immune response that results in hair loss. In addition, autoantibodies bind to antigens on the surface of hair follicle cells, forming immune complexes that further damage hair follicle cells. Immune system abnormalities in AA patients may also be related to other autoimmune diseases. For example, a study in 2021 found a higher incidence of thyroid dysfunction in AA patients compared to the general population, and when AA patients have thyroid dysfunction, the disease severity may be increased [31]. Thyroid disease is an autoimmune disease caused by immune system abnormalities, suggesting that AA may share similar pathogenesis with other autoimmune diseases. Another important factor is genetics. Studies have shown that the risk of AA in families of AA patients is several times higher than that in the general population, and about 10% of family members are diagnosed with AA [32]. Several genetic factors associated with AA have been identified, including the HLA gene cluster on chromosome 6. Specific HLA gene variants, such as *HLA-DQB1* and *HLA-DRB1*, are closely related to AA, whereas other non-HLA gene variants, such as *TLR7*, *IL-2/IL-21*, and *IL-23R*, are also associated with AA [33]. These studies indicate that genetic factors play an important role in the occurrence and development of AA. The pathogenesis of AA is complex, involving multiple cells, molecules, and pathological processes. Despite many related studies on the genetic level, unfortunately, the disease mechanism of AA is not fully understood.

## 4. Genetics of Alopecia Areata

### 4.1. Genetic Susceptibility

Although the pathogenesis of AA is multifactorial, numerous studies have confirmed that genetics plays an important role. A study on familial clustering of AA [32], conducted in Germany and Belgium, found that approximately 20% of patients had at least one family member with AA, indicating a genetic risk for AA. Moreover, AA tends to occur multiple times within the same family with a history of the disease, further underscoring its genetic basis. Other family and twin studies have confirmed the genetic risk for AA, which causes AA to occur more frequently among siblings than among parents and offspring [34]. Additionally, twin studies have provided valuable information on the genetic basis of AA, suggesting that the probability of both monozygotic twins being affected by AA is much higher than that of dizygotic twins both being affected if one of the twins is affected [35].

### 4.2. GWAS and Other Gene-Related Studies in Alopecia Areata

Petukhova et al. conducted the first genome-wide association study (GWAS) on AA, which identified 139 genotypes and 175 predicted single-nucleotide polymorphisms (SNPs) significantly associated with AA by comparing allele frequencies of 1054 unrelated AA patients and 3278 controls. These SNPs were mainly clustered in eight regions of the genome, covering immune-related and hair follicle-specific genes [36]. Lee et al. used exome sequencing to screen for candidate variants in six individuals with extensive alopecia universalis (AU) and identified 25 SNPs and 1 insertion/deletion. Subsequently, genotyping analysis of 14 additional AU patients revealed that six of these candidate variants were associated with AA or AU susceptibility [37]. Another GWAS study using pooling-based DNA genotyping found that variants in the HLA region showed the strongest association with AA, and the authors also identified the *SPATA5* gene locus as a novel susceptibility locus for AA [38].

In 2016, Petukhova et al. used three techniques, namely the identification of enriched pathways, biological processes, and protein–protein interactions (PPIs), to analyze the pathways associated with AA-related genes identified through GWAS. They found that the functions of these genes were associated with specific immune pathways, with the emphasis on the importance of the JAK-STAT signaling pathway, building the basis for future precision medicine development [39]. In addition, microRNAs have been implicated in AA. Aylar et al. [40] analyzed 617 microRNAs and found that 78 of them were significantly associated with AA, with miR-1237, miR-30b/d, and miR-548h-2 still being significantly associated with AA after correction. Among these, miR-30b/d was the most important microRNA in subsequent analyses due to its miRNA-specific signal in the regional association plot, significant expression in AA-related tissues, and predicted target genes that include several AA-associated loci. Thus, microRNA intervention may be a potential treatment strategy for AA in the future. Furthermore, another study in 2023 reported that the *KRT82* gene was significantly associated with AA. Among 849 AA patients, 19 patients (2.24%) had *KRT82* gene variants, while among 15,640 controls, only 88 people (0.56%) had *KRT82* gene variants. In addition, the study also identified two other genes, *KRTCAP3* and *DECR2*, that were associated with AA, but more studies are needed to confirm these findings [41]. In addition to GWAS, many studies, such as whole-exome sequencing and genome-wide microRNA analysis, have been conducted to evaluate the association between specific genes and AA in greater depth. We have reviewed the current genetic research related to AA and summarized the results in Table 4 and Table 5. Through GWAS analysis, whole-genome sequencing, and other analytical methods, genes associated with alopecia areata (AA) have been identified, mainly focusing on immune regulation genes, HLA genes, and inflammation-related pathway genes. Immune regulation genes such as *FASLG*, *PTPN22*, and *NOTCH4* are involved in T cell regulation and differentiation. Inflammation-related pathway genes such as *IL36A*, *IL-6*, and *IL-18* participate in cytokine release regulation, NF-κB and MAPK pathways, and coordination of immune function. Variations in HLA genes can affect immune cell recognition and attack hair follicles, leading to the development of AA. Genes such as *HLA-DRA*, *HLA-DRB*, and *HLA-DQA1* have been confirmed to be involved in the pathogenesis of AA. Other genes, such as *CLCNKA*, involved in regulating chloride ion transport across cell membranes, and *CPT2*, which encodes the carnitine palmitoyltransferase 2 protein involved in cellular fatty acid metabolism, have also been found to be associated with AA. Owing to the findings of genetic research, alopecia areata (AA) has been found to be associated with other diseases such as type 1 diabetes, rheumatoid arthritis, systemic lupus erythematosus (SLE), and atopic dermatitis. Table 5 summarizes the shared contributory genes between AA and other diseases. Therefore, during routine clinical evaluation of AA, physicians should also conduct comprehensive assessments for other diseases, including clinical inspection (atopic dermatitis), blood glucose levels (for type 1 DM), hormone levels (for Grave’s disease), immune-related markers (for SLE and multiple sclerosis), and gastrointestinal evaluations (for inflammatory bowel diseases, such as celiac disease and ulcerative colitis). These assessments aim to screen for potential comorbidities in AA patients. Since AA is visually apparent and easily detectable compared to the aforementioned autoimmune diseases, if an AA patient is found to have any of the autoimmune diseases listed in Table 5, the manifestation of AA can be considered an aiding biomarker for diagnosing the underlying immune disease. 

### 4.3. Relationship between Human Leukocyte Antigen (HLA) System and Alopecia Areata

The HLA (human leukocyte antigen) system is a critical component of the human immune system that is controlled by genes located on chromosome 6. Its primary function is to recognize and distinguish self cells from foreign cells and elicit an appropriate immune response to protect the body from invasion and harm by pathogens and foreign substances. Studies have shown that HLA-DRB1*04 and HLA-DRB1*16 polymorphisms are associated with increased AA risk, while HLA-DRB1*0301, HLA-DRB1*09, and HLA-DRB1*13 polymorphisms are associated with reduced AA risk [57]. Furthermore, a recent case-control study found an increased frequency of HLA-B*39 and HLA-DRB1*15 alleles in AA patients, while the frequency of HLA-A*11 and HLA-B*35 was lower [58]. Since AA is an autoimmune disease associated with an overactive immune system, studying the relationship between the HLA system and AA can advance our understanding of the disease mechanism, identify relevant pathogenic genes, and develop more effective treatment strategies.

### 4.4. Single Nucleotide Polymorphism (SNP) Studies

Genome-wide studies of genetic variants have led to the finding that single-nucleotide polymorphisms (SNPs) are involved in AA pathogenesis. A pilot study in 2022 showed that while *MTHFR* gene expression is significantly elevated in AA patients, variations in *MCP-1* rs1024611 and *MTHFR* rs1801133 may affect the pathogenesis of AA by impacting MCP-1 activity [70]. A study of Iranians analyzed the SNP genotypes of *FAS* (rs1800682) and *FASLG* (rs5030772) and found that the frequency of the G allele of *FASLG* gene is significantly higher in AA patients and there is an association between the *FASLG* rs5030772 variation and AA [42]. A study of Egyptians found a significant correlation between *MIR17HG* rs4284505 (A > G) and AA [63]. A case-control study of Jordanians analyzed some SNPs in five genes, *TAP1*, *CXCL1*, *CXCL2*, *HSPA1B*, and *TNFα*, and found a significant association between *TNFα* rs1800629 and AA [71]. Although SNP research in AA is increasing, the studies are still limited to specific populations. More extensive SNP research can help reveal the mechanism of AA occurrence, discover potential treatments, and provide new biomarkers for the prevention and diagnosis of AA. We summarize the SNP studies on AA in Table 6.

### 4.5. Studies of Gene Functions in Hair Follicles

Since it is well known that the pathogenesis of AA involves immune system attacks on hair follicles, gene functions in hair follicles may thus deserve further investigation. For example, Minjuan et al. found that the 3′ untranslated region (3′ UTR) of the junctional adhesion molecule A (*JAM-A*) gene functions as an important competitive endogenous RNA to maintain the function of hDPC, a specialized cell crucial for hair growth, and promote hair follicle regeneration in AA. JAM-A’s 3′ UTR forms a feedback loop with versican (*VCAN*) and miR-221-3p to regulate hDPC maintenance, proliferation, and differentiation [81]. Shymaa et al. [49] identified the involvement of hsa-miR-34a-5p in various hair follicle-related biological processes and vascular pathways by exploring the microRNA database. Their study revealed that *MIR34A* rs2666433 polymorphism and miR-34a may play a role in hair loss susceptibility. Furthermore, Syntaxin 17 (*STX17*) and Peroxiredoxin 5 (*PRDX5*) are genes related to the cellular and physiological functions of hair follicles. Mutations in *STX17* have been found to be associated with gray coat color in horses, and since dark-colored hair is more common in patients with AA, *STX17*, the gene involved in melanin synthesis, may thus be potentially associated with AA. In addition, *PRDX5* is thought to ameliorate cellular oxidative stress, the process often deregulated in the scalp of AA patients [50], and in AA patients’ hair follicle bulbs, the mRNA expression of Toll-like receptors 7 (*TLR7*) and interferon γ genes was significantly increased [53]. Together, understanding gene functions in hair follicles should lead to breakthrough discoveries in the treatment of AA and provide new directions for precision medicine.

## 5. Disease Associations with AA

Through the analysis in Table 4, we found that many of the genes associated with AA are also related to other diseases. As early as 2011, a nationwide population-based study revealed significant correlations between AA and vitiligo, systemic lupus erythematosus, psoriasis, atopic dermatitis, autoimmune thyroid disease, and allergic rhinitis [26]. In 2022, Sule Goksin et al. reported that AA patients not only tend to have autoimmune diseases but also develop multiple systemic diseases, autoimmune diseases, and psychiatric disorders [82]. Moreover, a study conducted in Saudi Arabia found that 62.7% of the 177 AA patients they recruited had insufficient serum vitamin D levels [83], which can also be observed in pediatric alopecia areata. A cross-sectional study found that pediatric alopecia areata patients tend to have comorbidities including atopic dermatitis, anemia, obesity, psoriasis, and depression. In summary, dermatologists treating AA patients need to have the ability to identify potential comorbidities and adopt a multidisciplinary approach to evaluate and manage these patients, thereby achieving better clinical outcomes.

## 6. Therapies for Alopecia Areata

### 6.1. Standard Management

Approximately 30–50% of patients diagnosed with patchy AA exhibit spontaneous remission within the initial six months to one year following disease onset, while approximately 60% of patients experience complete hair regrowth within a five-year timeframe [84]. Previous studies have indicated that patients with mild AA (scalp involvement area <25%) have demonstrated a spontaneous remission rate of approximately 68%. Furthermore, among patients with lesional involvement >50%, there is an observed spontaneous remission rate of 8% [85].

Although previous literature has indicated that certain cases of localized AA may undergo spontaneous resolution even without intervention, proactive management remains essential for patients with more severe presentations [86,87,88]. The current standard treatments for AA are commonly used worldwide, including in countries such as the United States, the United Kingdom, and Italy. We have compiled a consensus on AA treatment from various countries [87,89,90,91,92,93].

Intralesional corticosteroids (ICs) are the first-line treatment for small- to medium-sized areas of alopecia areata (AA) in adults, with Triamcinolone acetonide injection at concentrations of 2.5–10 mg/mL is commonly used. The main side effects of ICs are pitting atrophy and injection pain, which typically improve within a few months [33]. Topical corticosteroids (TCs) are the most common treatment for AA in patients who cannot receive ICs, with potent steroids such as betamethasone dipropionate 0.05% cream, lotion, or ointment being used in both adults and children [94]. Although occlusion may enhance treatment efficacy, there is insufficient evidence to prove its safety. Possible side effects include temporary folliculitis and skin atrophy. Systemic corticosteroids are also a common treatment, typically using an initial dose of oral prednisolone 0.5 mg/kg for a six-week course. However, long-term treatment may carry systemic side effects such as weight gain and risk of osteoporosis and a higher risk of relapse after discontinuation [94]. Minoxidil, a vasodilator, has been found to be a safe and effective treatment for AA when used locally in combination with other therapies. Studies have shown that low-dose minoxidil (0.25 to 5 mg daily to twice daily) is a safe and successful treatment for androgenetic alopecia and AA [95]. Methotrexate (MTX) is often used as monotherapy or adjuvant therapy for AA. A 2018 study found that combination therapy had a higher complete remission rate compared to monotherapy, but some patients experienced relapse after dose reduction [96]. Cyclosporine is also a commonly used treatment, and like MTX, it is often used in combination with other therapies. A systematic review by Joanna et al. [97] suggested that using cyclosporine in combination with systemic corticosteroids was more effective than using cyclosporine alone. In addition, diphenylcyclopropenone (DPCP) is the preferred drug for treating severe AA and regulates the autoimmune system by modulating the CD4+/CD8+ T lymphocyte ratio. A randomized clinical trial compared the efficacy of using DPCP alone versus combining DPCP with anthralin for treating AA. The study found that the combination of DPCP and anthralin had an effectiveness equal to that of DPCP alone, while the addition of anthralin did not enhance the therapeutic effect of DPCP in treating AA. [98]. Squaric acid dibutylester (SADBE) is another option for contact immunotherapy. A retrospective cohort study of 49 AA patients treated with SADBE in Japan confirmed that SADBE local immunotherapy is an effective and safe approach for treating AA [99]. Table 7 summarizes the standard treatments currently used in clinical practice for AA.

### 6.2. Novel Drugs for Alopecia Areata

In recent years, there has been a growing interest in emerging therapies for alopecia areata (AA), among which JAK inhibitors have received considerable attention. While further research is needed to establish the long-term safety of JAK inhibitors, several studies have already demonstrated their efficacy in treating AA [101]. Baricitinib, an orally administered Janus kinase 1 (JAK1) and JAK2 inhibitor, has been shown in phase 3 trials to effectively treat severe AA in adults without causing significant adverse effects [102]. In a phase 2 clinical trial involving 654 patients, they were randomly assigned to receive either 2 mg baricitinib, 4 mg baricitinib, or a placebo, administered once daily for 36 weeks. Compared to the placebo group, the group that received baricitinib had a significantly higher proportion of patients achieving the desired treatment goal (SALT ≤ 20) [103]. In a phase 3 trial involving 546 patients, similar treatment outcomes were observed, confirming the efficacy of baricitinib in improving the condition of AA patients [102]. Baricitinib was previously approved by the FDA in 2018 for the treatment of rheumatoid arthritis and is currently approved for the treatment of severe AA. Reported side effects of baricitinib include upper respiratory tract infections, headaches, acne, and others, but the severity is generally mild to moderate.

Ritlecitinib and brepocitinib have also shown positive results in two phase 2 trials for the treatment of AA [104]. A meta-analysis of JAK inhibitors [105] suggests that oral JAK inhibitors are effective for treating AA, but topical or sublingual administration may be less effective. Additionally, the efficacy of baricitinib, ritlecitinib, and brepocitinib appear to be similar in this study. Maddison et al. have provided a comprehensive review of JAK inhibitors for the treatment of AA, including other drugs such as tofacitinib, ruxolitinib, delgocitinib, and CTP-543 [106]. Although the efficacy of JAK inhibitors is supported by most studies, the high rate of relapse after discontinuation and the high cost of treatment still requires further investigation for long-term maintenance and drug performance. We compile the newer treatments for AA in Table 8.

In a 2019 randomized control trial study on patients with moderate to severe AA, Apremilast, a PDE-4 inhibitor, did not show significant improvement in the group receiving 30mg of oral Apremilast (*n* = 12) compared to placebo (*n* = 8) after 24 weeks of treatment [107]. Although this study yielded negative results, it suggests that the PDE-4 pathway may not be a therapeutic target for patients with moderate to severe AA. Further large-scale studies are needed to confirm the use of PDE-4 inhibitors in AA. Biologics such as Dupilumab, Aldesleukin, and Secukinumab have been studied for their use in AA treatment [108], but most studies have not shown significant therapeutic effects or have yielded conflicting results. Although biologics are a potential treatment option, there is currently no clear evidence to support their role in AA treatment.

In addition to above managements, Youyu et al. [109] analyzed 36 miRNAs with significantly different expressions in the blood of severe AA patients and found that miR-185-5p, miR-125b-5p, and miR-186-5p play important roles in active AA patients. These results suggest a correlation between miRNA expression in the blood and AA and may be used to predict disease progression or develop new treatment modalities in the future. In 2020, a study investigated the effects of long non-coding RNAs (lncRNAs) and competing endogenous RNAs (ceRNAs) on AA patients and found that specific ceRNAs may play a key role in the pathogenesis of AA by regulating the interaction between cytokines and cytokine receptors [110]. This finding suggests that ceRNAs may be a new target for future treatments. The use of plant-based preparations in hair loss has also been a topic of interest. Erkin et al. [111] used a topical mixture composed of six different herbal extracts (HE) for cell experiments and found that IL-1α gene expression was significantly decreased (*p* < 0.0001) in cells treated with HE. While IL-1α is a follicle growth inhibitor, this study thus provides a new idea for the treatment of hair loss using plant extracts. Mesenchymal stem cell therapy (MSCT) has also been proposed in several previous studies as a safe and effective treatment for difficult-to-treat AA [112,113]. Overall, there are currently many new treatment options for severe AA, and as our understanding of AA deepens, more new treatments are expected to emerge. However, even with new and effective treatment options, there is no guarantee that they will be applicable to all patients, and relapse rates are often high after treatment discontinuation [114]. Furthermore, AA is a systemic disease, and its associated comorbidities should be considered during treatment.

**Table 8:** Emerging treatments for alopecia areata (AA).

Therapy	Mechanism	Reference
Baricitinib	JAK1, 2/TYK2 inhibitor	[102]
Ritlecitinib	JAK 2 inhibitor	[115]
Brepocitinib	JAK1/TYK2 inhibitor	[115]
Tofacitinib	JAK1, 2, 3/TYK2 inhibitor	[116]
Ruxolitinib	JAK1, 2 inhibitor	[117]
Delgocitinib	JAK1, 2, 3/TYK2 inhibitor	[118]
CTP-543	JAK1, 2 inhibitor	[119]

## 7. Conclusions

This review summarizes recent genetic studies on AA and provides an overview of genome-wide association studies (GWAS) on AA in Table 4. Understanding the genetic structure and inheritance of AA can help elucidate the disease’s pathogenesis and develop effective treatments. Table 4 reveals that AA is linked to many immune-related genes, such as *PTPN22*, *HLA-DRA*, *HLA-DRB1*, and *AIRE*. However, recent studies have also found other genes with different physiological functions related to AA, such as *MT-ND1*, *COL6A2*, and *POLH*, suggesting that our understanding of AA remains incomplete. Although AA is known to be an autoimmune disease, its correlation with other diseases requires further investigation. Current research on AA has progressed to analyzing SNPs in different ethnic groups worldwide, which can help us understand the differences in alopecia rates between different populations and improve our understanding of the global prevalence of AA. We have compiled recent SNP studies in Table 5. Understanding subtle genetic variations can help identify susceptible populations and predict treatment responses. Breakthroughs in genetic research have led to the emergence of many promising treatment directions, such as JAK inhibitors, which have undergone late-stage clinical trials and proven to be effective for treating AA. In summary, genetic research plays an important role in understanding the pathogenesis and etiology of AA. With recent advances in research, multiple genes related to AA have been identified, and ongoing genetic research can lead to more effective and targeted treatments. However, due to the existence of multiple subtypes of AA and potential genetic and environmental differences between different ethnic groups, further research, and validation are necessary to establish a more comprehensive and accurate genetic database and related mechanisms, and provide more comprehensive support and help for the clinical diagnosis and treatment of AA.

## Figures and Tables

**Table 1:** Prevalence and incidence of AA according to epidemiological studies of different regions.

Study	Region	Incidence, %	Prevalence, %	Reference
2023, Mostaghimi	USA	0.0929	0.22	[2]
2023, Sy	USA	-	0.17	[11]
2023, Campos-Alberto	Japan	-	0.27	[12]
2022, Andersen	Denmark	-	0.9	[13]
2022, Harries	UK	0.026	0.2	[14]
2020, Alshahrani	Saudi Arabia	-	2.3	[15]
2020, Abdulmajed	Saudi Arabia	-	5.25	[16]
2020, Benigno	USA	-	2.51	[17]
2020, Lee	Chicago	1.7	2.13	[18]
2019, Soh	South Korea	0.985	0.15	[19]
2014, Mirzoyev	USA	2.1	-	[20]
2007, Guzmán-Sánchez	Mexico	0.57	-	[21]
2002, Tan	Singapore	3.8	-	[22]
1996, Sharma	India	0.7	-	[23]
1995, Safavi	USA	1.7	-	[24]
1991, Price	USA and Britain	2	-	[25]

**Table 2:** Prevalence of AA by age group according to epidemiological studies.

		Age Groups *								Reference
Study	Region	0–9	10–19	20–29	30–39	40–49	50–59	60–69	70–79	
2023, Sy	USA	0.07	0.13	0.16	0.28	0.28	0.18	0.16	0.15	[11]
2023, Mostaghimi	USA	0.07	0.17	0.28	0.24		-	0.17	-	[2]

* Approximate prevalence of patients with AA in each age group.

**Table 3:** The average age of diagnosis/Onset of alopecia areata globally.

Study	Region	Age of Diagnosis/Onset	Reference
2020, Alshahrani	Saudi Arabia	25.6	[15]
2020, Abdulmajed	Saudi Arabia	19	[16]
2013, Mirzoyev	USA	33.6	[20]
2011, Chu	Taiwan	32.2	[26]
2006, Goh	USA	36.3	[27]
2002, Tan	Singapore	25.2	[22]
2004, Yang	China	29	[28]

**Table 4:** Genes with strong association with alopecia areata.

Location	Gene	Associated Function	Connection to Other Diseases *	Reference
Chromosome 1	*FASLG*	Activation-induced cell death (AICD) of T cells	Not defined	[42]
	*PTPN22*	Regulating CBL function in the T-cell receptor signaling pathway	T1D, RA, SLE, GD	[43,44,45]
	*CLCNKA*	Salt reabsorption in the kidney and potassium recycling in the inner ear	Bartter syndrome, type 4b	[46]
	*CLCNKB*	Renal salt reabsorption	Not defined	[46]
	*CPT2*	Oxidization of long-chain fatty acids in the mitochondria	Carnitine palmitoyltransferase II deficiency, infantile	[46]
	*PINK1*	Protection of cells from stress-induced mitochondrial dysfunction	PD	[46]
	*SUCO*	Collagen biosynthetic process	Mesial temporal lobe epilepsy	[46]
	*USH2A*	Development and homeostasis of the inner ear and retina	Deafness	[46]
	*MASP2*	Coagulation cascade	Not defined	[47]
	*CD2*	Immune recognition	Not defined	[48]
	*MIR34A*	Tumor suppressor	Not defined	[49]
Chromosome 2	*CTLA4*	Co-stimulation	T1D, RA, CeD, MS, SLE, GD	[5,39,45,47,50,51]
	*ICOS*	Co-stimulation	T1D, MS	[5,39,50]
	*ACOXL/* *BCL2L11*	Apoptosis, autophagy regulation	T1D, IgA nephropathy, primary sclerosing cholangitis	[5]
	*IL36A*	Inflammatory response	Allergic contact dermatitis, AD, acne, hidradenitis suppurativa	[52]
	*ALS2*	A guanine nucleotide exchange factor for the small GTPase RAB5	ALS	[46]
	*CYP27A1*	Drug metabolism and synthesis of cholesterol, steroids, and other lipids	CTX, Cholestanol storage disease	[46]
	*IRS1*	Encoding a protein that is phosphorylated by insulin receptor tyrosine kinase	DM	[46]
	*COL4A4*	The structural component of basement membranes	Thin basement membrane disease	[46]
	*TPO*	Thyroid gland function	Congenital hypothyroidism	[46]
	*HOXD13*	Morphogenesis in all multicellular organisms	Synpolydactyly	[46]
Chromosome 3	*CHRNB2*	Regulation of synaptic vesicle exocytosis	AD	[46]
	*COLQ*	Encoding the subunit of a collagen-like molecule	Congenital myasthenic syndrome	[46]
	*TLR9*	Pathogen recognition and activation of innate immunity	Not defined	[53]
Chromosome 4	*IL-21*	Th17 and NK cell proliferation	T1D, RA, CeD, PS	[5,39,50]
	*IL-2*	T and B cell proliferation	T1D, RA, CeD, PS	[5,39,50]
	*Cxcl9*	Chemoattractant for lymphocytes	RA, T1D, PS, SLE	[5]
	*Cxcl10*	Chemokine: monocyte, NK and, T cell stimulation	RA, T1D, PS, SLE	[5]
	*Cxcl11*	Chemokine: chemotaxis of activated T cells	RA, T1D, PS, SLE	[5]
	*TLR1*	Pathogen recognition and activation of innate immunity	Not defined	[47,50]
	*EGF*	Growth, proliferation, and differentiation of numerous cell types	Not defined	[54]
Chromosome 5	*IL-13/IL-4*	Th2 differentiation	Not defined	[5,50]
	*IL12B*	Mediation of long-term protection to an intracellular pathogen	MS	[55]
	*IL7R*	V(D)J recombination during lymphocyte development	PIDDs	[46]
	*IL31RA*	Type I cytokine receptor family	Not defined	[56]
	*VCAN*	Tissue morphogenesis and maintenance	Retinitis pigmentosa	[46]
Chromosome 6	*NOTCH4*	T cell differentiation	T1D, RA, MS	[5]
	*C6orf10*	Unknown function in vivo	T1D, RA, PS, GV	[5,50]
	*BTNL2*	Co-stimulation	T1D, RA, UC, CD, SLE, MS	[5,50]
	*HLA-DRA*	Antigen presentation (MHC II)	T1D, RA, CeD, MS, GV	[5,50]
	HLA-DRB1*04	Antigen presentation (MHC II)	Not defined	[57]
	HLA-DRB1*16	Antigen presentation (MHC II)	Not defined	[57]
	HLA-DRB1*11	Antigen presentation (MHC II)	Not defined	[57]
	*HLA-DQA1*	Antigen presentation (MHC II)	T1D, RA, UC, CD, SLE, MS, CeD, GD	[5,50]
	*HLA-DQA2*	Antigen presentation (MHC II)	T1D, RA	[5,50]
	*HLA-DQB2*	Antigen presentation (MHC II)	RA	[5,50]
	*HLA-DOB*	Antigen presentation (MHC II)	SLE	[5]
	*HLA-A*	Antigen presentation (MHC I)	T1D, MS, PS, GD	[50]
	HLA-B*13	Antigen presentation (MHC I)	Not defined	[58]
	*KLRK1*	NK and T cell activation (NKG2D)	T1D, RA, MS, CD, CeD, SLE	[5]
	*MICA*	NKG2D Activating ligand	T1D, RA, UC, CeD, PS, SLE	[5,50]
	*ULBP6*	NKG2D Activating ligand	Not defined	[5,50]
	*ULBP3*	NKG2D Activating ligand	Not defined	[5,50]
	*TNFA*	Proinflammatory cytokine	RA, MS, IBD, SLE	[5]
	*PPP1R18*	Targeting the enzyme to different cellular locations	Not defined	[40]
	*TNXB*	Localizing to the major histocompatibility complex (MHC) class III	Not defined	[40]
	*POLH*	A member of the Y family of specialized DNA polymerases	XP, variant type	[46]
	*COL9A1*	Assembly of type IX collagen molecules	Not defined	[46]
Chromosome 7	*IL-6*	Inflammatory cytokine	T1D, RA, CeD	[5]
	*SLC26A4*	No known	Enlarged vestibular aqueduct, Pendred’s syndrome	[46]
	*EGFR*	Protein kinase superfamily	Lung cancer, severe form of coronavirus disease 2019 (COVID-19)	[54]
Chromosome 8	*LPL*	Encodes lipoprotein lipase	Hyperlipoproteinemia, type I	[46]
Chromosome 9	*STX17*	No known inflammatory role. It is involved in premature hair greying	Not defined	[5,50]
	*GNE*	Initiates and regulates the biosynthesis of N-acetylneuraminic acid (NeuAc)	Inclusion body myopathy 2	[46]
Chromosome 10	*IL-2RA*	T-cell proliferation	T1D, MS, GD, GV	[5,45,50]
	*TWNK*	mtDNA replication	Ataxia	[46]
	*DKK1*	Embryonic development	Not defined	[59]
	*TCF7L2*	Wnt signaling pathway	DM	[60]
Chromosome 11	*PRDX5*	Antioxidant enzyme with roles in inflammation	MS	[5,50]
	*IL-18*	Proinflammatory cytokine that augments natural killer cell activity and stimulates IFNγ production in T-helper type I cells	RA, SLE	[5,61]
	*GARP (LRRC32)*	Treg differentiation and activity	IBD, Allergies	[5]
	*SLC22A12*	Regulation of urate levels in blood	Renal hypouricemia	[46]
	*TYR*	Encoding tyrosinase	Oculocutaneous albinism	[46]
Chromosome 12	*IL-26*	T cell differentiation	MS	[5]
	*IFNG*	Regulation of immune responses	SLE	[5]
	*KRT82*	A member of the keratin gene family	Not defined	[41]
	*CD27*	T cell immunity and regulating B-cell activation	Not defined	[62]
	*WNT10B*	Implicated in oncogenesis and in several developmental processes	Oligodontia	[46]
Chromosome 13	*MIR17HG*	Cell survival, proliferation, differentiation, and angiogenesis	Not defined	[63]
Chromosome 15	*IL16*	The modulator of T cell activation and an inhibitor of HIV replication	Not defined	[64]
	*CHAC1*	Promotion neuronal differentiation	Not defined	[65]
Chromosome 16	*SOCS1*	STAT inhibitor, regulator of IFN-γ response	T1D, CeD	[5]
	*FUS*	Regulation of gene expression, maintenance of genomic integrity, and mRNA/microRNA processing	ALS	[46]
Chromosome 17	*CCL13*	Chemotactic activity for monocytes, lymphocytes, basophils, and eosinophils	Not defined	[66]
Chromosome 18	*PTPN2*	Phosphatase involved in cell signaling	T1D, CeD	[5]
Chromosome 19	*CD70*	Enhances T helper and cytotoxic T cell activation	Not defined	[62]
	*NPHS1*	Ultrafilter to exclude albumin and other plasma macromolecules in the formation of urine	Finnish congenital nephrotic syndrome	[46]
Chromosome 20	*PIGT*	Glycosylphosphatidylinositol (GPI)-anchor biosynthesis	Multiple congenital anomalies-hypotonia- seizures syndrome 3	[46]
	*RTEL1*	Encodes a DNA helicase	Dyskeratosis congenita	[46]
Chromosome 21	*AIRE*	Autoimmune regulator, selection of auto-reactive cells	APECED, T1D, GV, HT	[5]
	*COL6A2*	Encodes one of the three α chains of type VI collagen	Muscular dystrophy	[46]
Chromosome 22	*CELSR1*	Cell adhesion and receptor-ligand interactions	Neural tube defects	[46]
	*IL17RA*	Inducer of the maturation of CD34-positive hematopoietic precursors into neutrophils.	RA	[56]
Chromosome X	*Cxcr3*	Chemokine receptor	RA, T1D, PS, SLE	[5]
	*MAMLD1*	Transcriptional co-activator	46XY disorder of sex development	[46]
	*TLR7*	Pathogen recognition and activation of innate immunity.		[53]
Chromosome MT	*MT-ND1*	Mitochondrial electron transport	AD, PD	[46]

* Abbreviation: Type 1 diabetes (T1D), rheumatoid arthritis (RA), systemic lupus erythematous (SLE), Graves’ disease (GD), Parkinson’s disease (PD), celiac disease (CeD), multiple sclerosis (MS), amyotrophic lateral sclerosis (ALS), cerebrotendinous xanthomatosis (CTX), psoriasis (PS), primary immunodeficiency diseases (PIDDs), generalized vitiligo (GV), ulcerative colitis (UC), Crohn’s disease (CD), inflammatory bowel disease (IBD), xeroderma pigmentosum (XP), Hashimoto thyroiditis (HT).

**Table 5:** Genes associated with both alopecia areata and other diseases.

Disease	Genes	Reference
Type 1 Diabetes	*PTPN22, CTLA4, ACOXL/BCL2L11, IL-21, IL-2, Cxcl9, Cxcl10, Cxcl11, NOTCH4, C6orf10, BTNL2, HLA-DRA, HLA-DQA1, HLA-DQA2, HLA-A, KLRK1, MICA, IL-6, IL-2RA, SOCS1, PTPN2, Cxcr3*	[5,50]
Rheumatoid Arthritis	*PTPN22, CTLA4, IL-21, IL-2, Cxcl9, Cxcl10, Cxcl11, NOTCH4, C6orf10, BTNL2, HLA-DRA, HLA-DQA1, HLA-DQA2, HLA-DQB2, KLRK1, MICA, TNFA, IL-6, IL-18, IL17RA, Cxcr3*	[5,50]
Systemic Lupus Erythematous	*PTPN22, CTLA4, Cxcl9, Cxcl10, Cxcl11, BTNL2, HLA-DQA1, HLA-DOB, KLRK1, MICA, TNFA, IL-18, IFNG, Cxcr3*	[5,50]
Graves’ Disease	*PTPN22, CTLA4, HLA-DQA1, HLA-A, IL-2RA,*	[5,50]
Parkinson’s Disease	*PINK1, MT-ND1*	[5,46]
Celiac Disease	*CTLA4, IL-21, IL-2, HLA-DRA, HLA-DQA1, KLRK1, MICA, IL-6, SOCS1, PTPN2*	[5,50]
Ulcerative Colitis	*BTNL2, HLA-DQA1, MICA,*	[5]
Multiple Sclerosis	*CTLA4, ICOS, IL12B, NOTCH4, BTNL2, HLA-DRA, HLA-DQA1, HLA-A, KLRK1, TNFA, IL-2RA, PRDX5, IL-26*	[5,50,55]
Amyotrophic Lateral Sclerosis	*ALS2, FUS*	[5,46]
Generalized Vitiligo	*C6orf10, HLA-DRA, IL-2RA, AIRE*	[5]
Inflammatory Bowel Disease	*TNFA, GARP (LRRC32)*	[5]
Atopic Dermatitis	*HLA-DQB1, HLA-DRB1, HLA-DQA1, IL-4, IL-13, IL-17, IL-23R*	[67,68,69]

**Table 6:** Associated-SNP studies with Alopecia Areata.

Study	Population	Gene	SNP	Finding	Reference
2022, Alghamdi	Not defined	*IL17RA*	rs879575	Not associated with AA susceptibility among Jordanian patients	[56]
		*IL31RA*	rs161704		
2021, Gil-Quiñones	Not defined	*PTPN22*	rs2476601	T allele is a risk factor for developing AA	[45]
2021, Conteduca	Italian	*ICOS*	rs4404254 rs4675379	Carrying the 3’ UTR alleles was more frequently observed in AA patients	[72]
2021, Ismail	Egyptian	*CTLA4*	rs231775	Significantly higher in AA patients	[51]
2020, Abd El-Raheem	Egyptian	*TNFα* promoter region	rs1800629	No association with AA	[73]
2020, Eitan	Jordanian	*IL16* exon region	rs11073001	The A-allele was distributed more frequently	[64]
		*IL16* promoter region	rs17875491	A difference was found between the patients and the controls	
2019, Lei	Not defined	*PTPN22*	rs2476601	Significantly correlated with AA. The C-allele and CC-genotype carriers at this locus have a lower risk of AA.	[74]
2019, Al-Eitan	Jordanian	*TNFα*	rs1800629	Significantly associated with AA in the heterozygous and rare homozygous genotypes	[71]
2018, Sumeyya	Turkish	*IL 18*	rs1946518	Distribution of CC + CA genotypes and frequency of -607/allele C were higher in AA	[61]
			rs187238	Distribution of GG genotype and frequency of -137/allele G were higher in AA	
2015, Kalkan	Turkish	*MnSOD*	Ala-9Val	No association with AA	[75]
			GPx1 Pro 198 Leu	No association with AA	
2015, Kim	Korean	*TAP1* promoter region	rs2071480	Association with AA	[76]
2015, Salinas-Santander	Mexican	*PTPN22*	C1858T	T allele as a genetic risk factor for patchy AA	[44]
2014, Conteduca	Not defined	*FOXP3*	rs2294020	Reduced relative gene expression in AA patients	[77]
		*ICOSLG*	rs378299		
2014, Kim	Korean	*IL18*	rs187238 rs549908	Associated with the development of AA	[78]
2014, Seok	Korean	*TLR1* missense region	rs4833095	Significantly associated with the development of AA	[79]
		*TLR1* promoter region	rs5743557	Weakly associated with the development of AA	
2014, Seok	Korean	*HSPA1B*	rs6457452	Weakly related to the age of onset of AA	[80]
			rs2763979	Weakly related to AA	
2012, Forstbauer	European	*SPATA5* intronic region	rs304650	Significant association with AA	[38]

**Table 7:** Standard treatments for alopecia areata (AA).

Therapy	Administration	Side Effects	Reference
Intralesional corticosteroids	Topical Injected directly into the skin	Pain, skin atrophy, contact allergic dermatitis	[88,94,100]
Topical corticosteroids	Topical foam or cream	Folliculitis, skin atrophy, acne, telangiectasia	[88,94,100]
Systemic corticosteroids	Oral intake or intravenous or intramuscular injection	Suppression of the pituitary–adrenal axis, weight gain, osteoporosis, ocular changes hypertension, diabetes	[88,94,100]
Topical minoxidil	Topical foam or cream	Itching and dermatitis, hypertrichosis, acne	[88,94,100]
Methotrexate	Oral intake alone or in conjunction with corticosteroids	Nausea and vomiting, mucositis, liver toxicity, leukopenia	[88,94]
Cyclosporine	Oral intake alone or in conjunction with corticosteroids	Hypertension, hypertrichosis, nephrotoxicity	[88,94]
Contact immunotherapy diphenylcyclopropenone (DPCP) squaric acid dibutylester (SADBE)	Topical cream	Lymphadenopathy, generalized eczema, vitiligo	[88,94,100]

## Data Availability

Not applicable.
